# *NF1* microdeletion syndrome: case report of two new patients

**DOI:** 10.1186/s13052-019-0718-7

**Published:** 2019-11-08

**Authors:** Gregorio Serra, Vincenzo Antona, Giovanni Corsello, Federico Zara, Ettore Piro, Raffaele Falsaperla

**Affiliations:** 1 Department of Sciences for Health Promotion and Mother and Child Care “G. D’Alessandro”, University of Palermo, Palermo, Italy; 2 Laboratory of Neurogenetics and Neuroscience, Institute G. Gaslini, Genoa, Italy; 3 Unit of Pediatrics and Pediatric Emergency, University Hospital “Policlinico-Vittorio Emanuele”, Catania, Italy

**Keywords:** *NF1* gene, Atypical deletion, Genotype-phenotype correlation, Contiguous gene syndrome, MLPA

## Abstract

**Background:**

17q11.2 microdeletions, which include the neurofibromatosis type 1 (*NF1*) gene region, are responsible for the *NF1* microdeletion syndrome, observed in 4.2% of all NF1 patients. Large deletions of the *NF1* gene and its flanking regions are associated with a more severe NF1 phenotype than the NF1 general population.

**Case presentation:**

We hereby describe the clinical and molecular features of two girls (aged 2 and 4 years, respectively), with non-mosaic atypical deletions. Patient 1 showed fifteen *café-au-lait* spots and axillary freckling, as well as a Lisch nodule in the left eye, strabismus, high-arched palate, malocclusion, severe kyphoscoliosis, bilateral calcaneovalgus foot, mild generalized hypotonia, hyperactivity and deficits of speech-related abilities. *NF1* genomic rearrangements through multiplex ligation-dependent probe amplification (MLPA) detected an heterozygous deletion of the whole *NF1* gene. Array comparative genomic hybridization (a-CGH) analysis defined a 17q11.2 deletion of about 1 Mb (breakpoints at positions 29,124,299 and 30,151,654), which involved different genes (partially *CRLF3*, *ATAD5*, *TEFM*, *ADAP2*, *RNF135*, *OMG*, *EVI2B*, *EVI2A*, *RAB11FIP4*), including *NF1*. Patient 2 showed growth and developmental delay, supravalvular pulmonary stenosis, twenty-five *café-au-lait* spots, axillary freckling, craniofacial dysmorphic features, short neck with *pterygium*, limb abnormalities and *foci* of neural dysplasia on brain magnetic resonance imaging (MRI). MLPA detected an heterozygous deletion of *NF1*, which was detailed by a-CGH indicating the positions 29,124,299 and 30,326,958 as its breakpoints, and which included aside from the genes deleted in Patient 1 also *COPRS*, *UTP6* and partially *SUZ12*. Fluorescent in situ hybridization (FISH) analysis of the parents documented a de novo origin of the deletions in both cases.

**Conclusions:**

The present report will likely provide further insights and a better characterization of *NF1* microdeletion syndrome.

## Background

Neurofibromatosis type 1 (NF1; MIM#162200) is a neurogenetic disorder with a birth prevalence estimated around 1:2500 [1]. NF1 shows an autosomal dominant pattern of inheritance and wide phenotypical variability. *Café-au-lait* spots (CALs), cutaneous and/or subcutaneous neurofibromas (CNFs/SCNFs), skinfold freckling, skeletal abnormalities and Lisch nodules of the iris are its main clinical features. NF1 patients have an increased risk of learning and intellectual disabilities as well as tumors of the nervous system and other organs [1]. *NF1* gene maps on chromosome 17q11.2. Microdeletions of this region are responsible for *NF1* microdeletion syndrome, observed in 4.2% of all NF1 patients. Large deletions of *NF1* and its flanking regions have been associated with more severe phenotype than NF1 general population. Four types (1, 2, 3 and atypical) of such large *NF1* deletions have been reported so far, with differences in size, breakpoint location, number of genes deleted and somatic mosaicism [2]. Type-1 is the most frequent (70–80%), while 8–10% are atypical [3]. In the present study we report on the clinical and molecular features of two patients with non-mosaic atypical *NF1* deletions, in order to give further insights and a better characterization of *NF1* microdeletion syndrome.

## Case presentation

### Patient 1

**Fig. 1:**
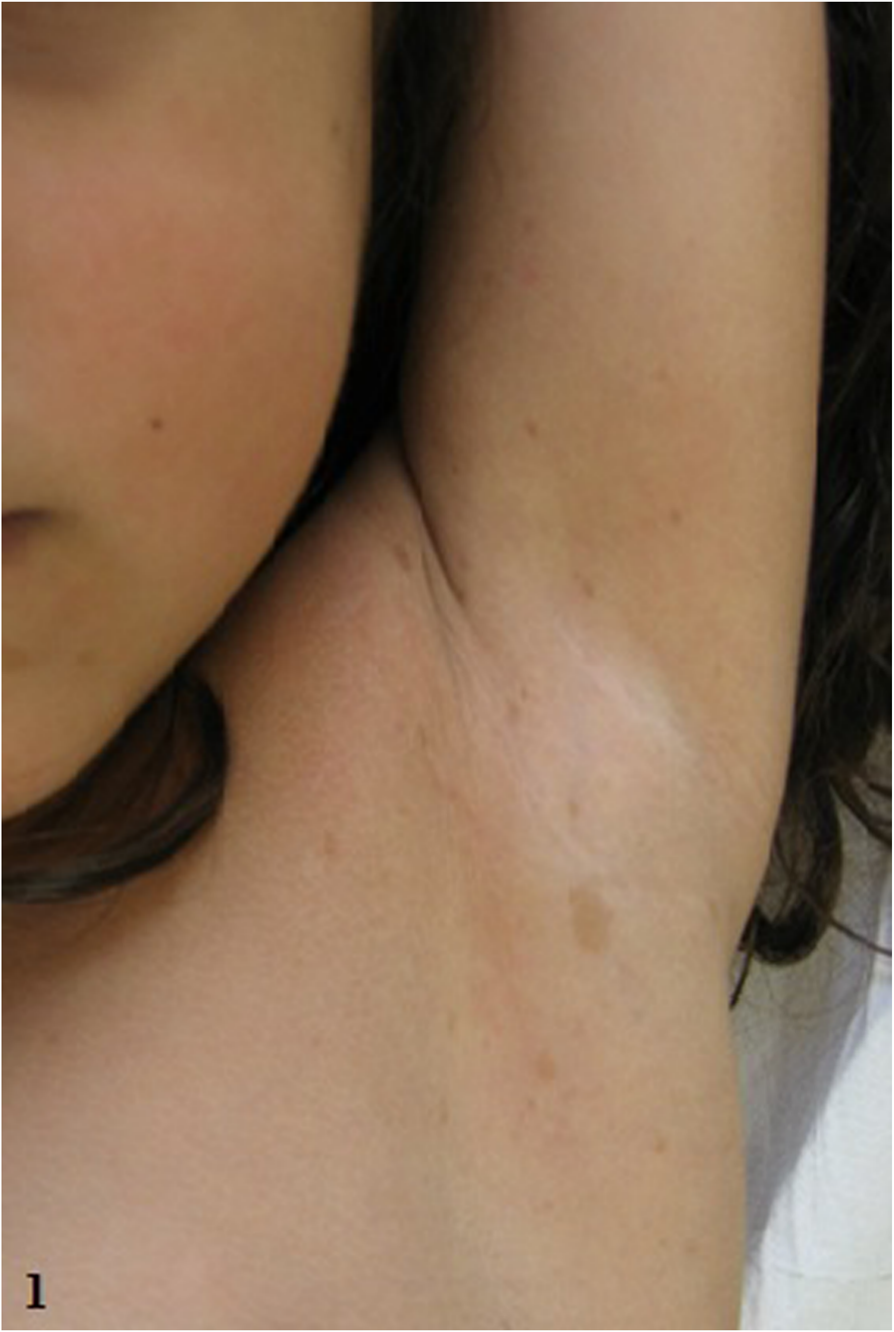
Patient 1: axillary frackling

This 4-year-old female was the first born, at 35 weeks of gestation, by vaginal delivery. Family and pregnancy histories were uneventful. At birth her weight was 2400 g (62nd percentile), length 45 cm (46th percentile), and occipitofrontal circumference (OFC) 31.3 cm (41st percentile). At the time of the presentation, her weight was 14.700 kg (25th percentile), height 104 cm (50th to 75th percentile) and OFC 49.5 cm (25th to 50th percentile). She had fifteen *café-au-lait* spots (mainly located in the nuchal and gluteal regions, the left elbow and the posterior surface of the right lower limb) and axillary freckling (Fig. 1), as well as a Lisch nodule in the left eye, strabismus, high-arched palate and malocclusion. Severe kyphoscoliosis and bilateral calcaneovalgus foot were also noted.

The neuropsychiatric evaluation, by Wechsler Preschool and Primary Scale of Intelligence (WPPSI) [4] and Peabody Picture Vocabulary Test-III (PPVT-III), showed deficits of speech-related abilities (Table 1).

**Table 1:** Neuropsychiatric evaluation. Patient 1 profile has been evaluated by WPPSI-IV (Wechsler Preschool and Primary Scale of Intelligence for children younger than 6 yr of age)

WPPSI-IV (Wechsler Intelligence Scale for children)	
Total IQ	88 (within normal value)
Verbal IQ	71 (low value)
Performance IQ	122 (within normal value)

Mild generalized hypotonia, dysgraphia (she went to primary school and was evaluated after the full school reading, writing and calculation skills attained by the first four months) and hyperactivity completed her clinical profile at the age of 6. Brain MRI and US heart evaluation showed no abnormalities.

No pathogenic mutations were revealed by molecular analysis of the *NF1* and *SPRED1* genes, through amplification of the coding regions and the flanking intronic sequences and sequencing of the amplified regions. Then, *NF1* genomic rearrangements through multiplex ligation-dependent probe amplification (MLPA) were performed, and the heterozygous deletion of the whole *NF1* gene was detected. Array comparative genomic hybridization (a-CGH) analysis (100–150 Kb resolution, genomic assembly GRCh37.p13) defined a 17q11.2 deletion of about 1 Mb, and identified the breakpoints at positions 29,124,299 and 30,151,654. The deleted region involved different genes (partially *CRLF3*, *ATAD5*, *TEFM*, *ADAP2*, *RNF135*, *OMG*, *EVI2B*, *EVI2A*, *RAB11FIP4*), including *NF1*. The rearrangement was confirmed by fluorescent in situ hybridization (FISH) (Additional file 1).

### Patient 2

This 2-year-8-month-old female was the second born by vaginal delivery, from healthy non-consanguineous parents, at 37^+ 5^ weeks of gestation after an uneventful pregnancy. At birth her weight was 2860 g (34th percentile), length 45 cm (4th percentile), OFC 31 cm (3rd percentile). At 8 months of age her weight, length and OFC were 6670 g (3rd to 10th percentile), 66 (3rd to 10th percentile) and 42 cm (3rd to 10th percentile), respectively. At her first neurodevelopmental examination at 18 months old, a global developmental delay was observed. EEG was normal, while US heart evaluation showed mild supravalvular pulmonary stenosis (pressure gradient <50 mmHg).

At the age of 2, because of her growth delay, cardiomyopathy and dysmorphic features, sequencing of the genes *PTPN11*, *RAF1*, *BRAF1*, *MEK1/2*, *KRAS*, *SOS1* and *SHOC2* were performed. No abnormalities were detected. Plasma amino acid pattern, plasma and urine levels of glycosaminoglycans, acylcarnitine profile and urine organic acids were normal.

**Fig. 2:**
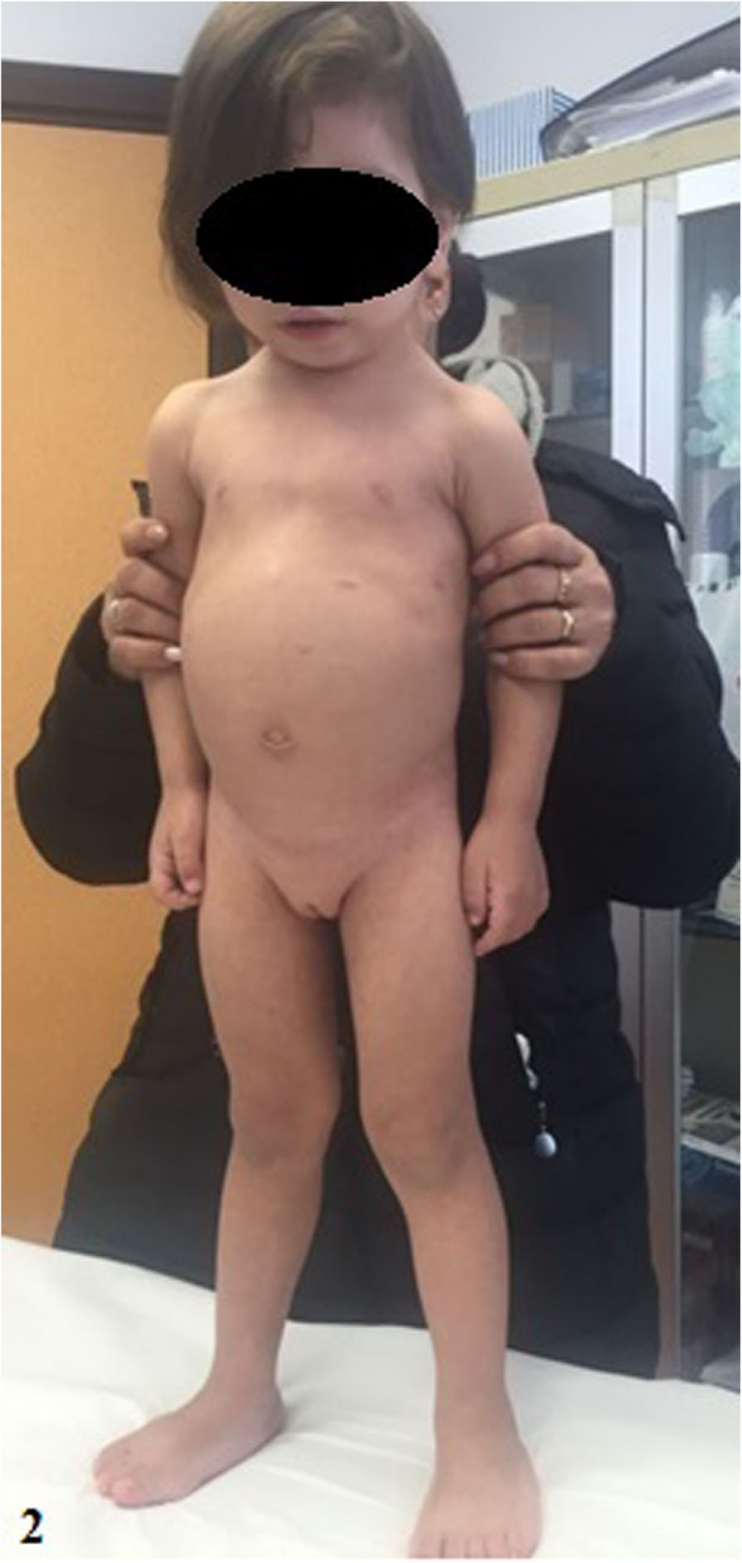
Patient 2: note *café-au-lait* spots, *pectus excavatum*, wide-spaced nipples, *diastasis* *recti abdominis* with prominent abdomen and bilateral *genu valgum* and *pes plano-valgus*

At the time of the presentation, her weight was 10.160 kg (<10th percentile), height 83 cm (<10th percentile) and OFC 47.3 cm (<10th percentile). She had twenty-five *café-au-lait* spots (mainly located in the anterior thoracic-abdominal region, upper back, posterior surface of the left thigh and proximal extremity of the right lower limb) and axillary freckling, as well as craniofacial dysmorphic features (broad forehead, dysplasic and low-set ears with thick helix, synophris, receding orbital roof with exophthalmos, hypertelorism, depressed nasal bridge, bulbous nose, malar hypoplasia, long and prominent philtrum, thick lips) and short neck with *pterygium*. *Pectus excavatum*, wide-spaced nipples, supernumerary areola, *diastasis recti abdominis* with prominent abdomen were also noted (Fig. 2). Short hands and feet, clinodactyly of the 5th finger, deep palmar creases and bilateral *genu valgum* and *pes plano-valgus* were observed (Fig. 2). Brain MRI revealed corpus callosum hypoplasia, T2 hyperintensities near the fourth ventricle, periventricular hyperintensities and a hyperintense nodule in the left thalamus, which were thought as *foci* of neural dysplasia with defect of myelination.

During a follow-up visit her cognitive profile was evaluated by WPPSI-IV for children younger than 6 yr of age, leading to the diagnosis of a mild intellectual disability (Table 2).

**Table 2:** Neuropsychiatric evaluation**.** Patient 2 profile has been evaluated by WPPSI-IV (Wechsler Preschool and Primary Scale of Intelligence for children younger than 6 yr of age)

WPPSI-IV (Wechsler Intelligence Scale for children)	
Total IQ	55 (low value)
Verbal IQ	64 (low value)
Performance IQ	61 (low value)

Molecular analysis of the genes *NF1* and *SPRED1*, through amplification of the coding regions and the flanking intronic sequences and sequencing of the amplified regions did not detect any pathogenic mutations. Therefore, *NF1* genomic rearrangements through MLPA were performed, and identified an heterozygous deletion of *NF1*. a-CGH (100–150 Kb resolution, genomic assembly GRCh37.p13) detailed a deletion of 1.2 Mb, and indicated the positions 29,124,299 and 30,326,958 as the breakpoints of the deletion, which included aside from the genes deleted in Patient 1 also *COPRS*, *UTP6* and partially *SUZ12*. FISH was performed in both subjects aside from peripheral blood leucocytes also on buccal swab DNA, confirming the rearrangement also in peripheral tissue and ruling out somatic mosaicism (Additional file 1).

### Familial gene analysis

In both children and their parents, all exonic and intronic regions of *NF1* gene and its flanking regions were amplified through polymerase chain reaction (PCR), and the products were purified and directly sequenced. FISH analysis revealed in both cases a de novo origin of rearrangements.

## Discussion and conclusions

17q11.2 microdeletions, encompassing *NF1* gene, are responsible for *NF1* microdeletion syndrome [5], which include dysmorphic features, neurodevelopmental delay, cardiovascular defects, overgrowth, a higher tumor burden and earlier onset of benign neurofibromas and malignant peripheral nerve sheath tumors (MPNST)/other malignancies [2]. It represents 4.2% of all NF1 patients. The first 17q11.2 microdeletion patient was reported in 1992. Since then, more than 150 subjects have been described [6].

Four types of large *NF1* deletion have been identified (1, 2, 3 and atypical), recognizable by size, breakpoint location, number of genes deleted and somatic mosaicism [2].

Type-1 *NF1* deletion patients, whose anomaly encompasses 1.4 Mb and comprises 14 protein-coding genes and 4 microRNA genes, are the most frequent (70–80%). Type-2 extends for 1.2 Mb and depends on hemizygosity of 13 genes, sparing *LRRC37B* (about 10%). Type-3 is very rare (1–4%), spanning 1.0 Mb for a total of 9 genes [3]. Atypical deletions do not show recurrent breakpoints and are heterogeneous in size and genes deleted (8–10%) [2]. Unlike the well documented clinical phenotype of type-1 *NF1* deletions, a more detailed characterization of patients with atypical *NF1* deletions is lacking.

Here we have presented two de novo non-mosaic atypical deletions detected by MLPA, and defined through a-CGH, in two girls with developmental delay and dysmorphic features of variable clinical expressivity and severity.

No pathogenic mutations were firstly revealed by molecular analysis of the *NF1* and *SPRED1* genes, through amplification of the coding regions and the flanking intronic sequences and sequencing of the amplified regions. However, these tests do not allow the identification of genomic rearrangements like gain or losses of genetic material. Therefore, *NF1* genomic rearrangements through multiplex ligation-dependent probe amplification (MLPA) were performed, and the heterozygous deletion of the whole *NF1* gene was detected.

**Fig. 3:**
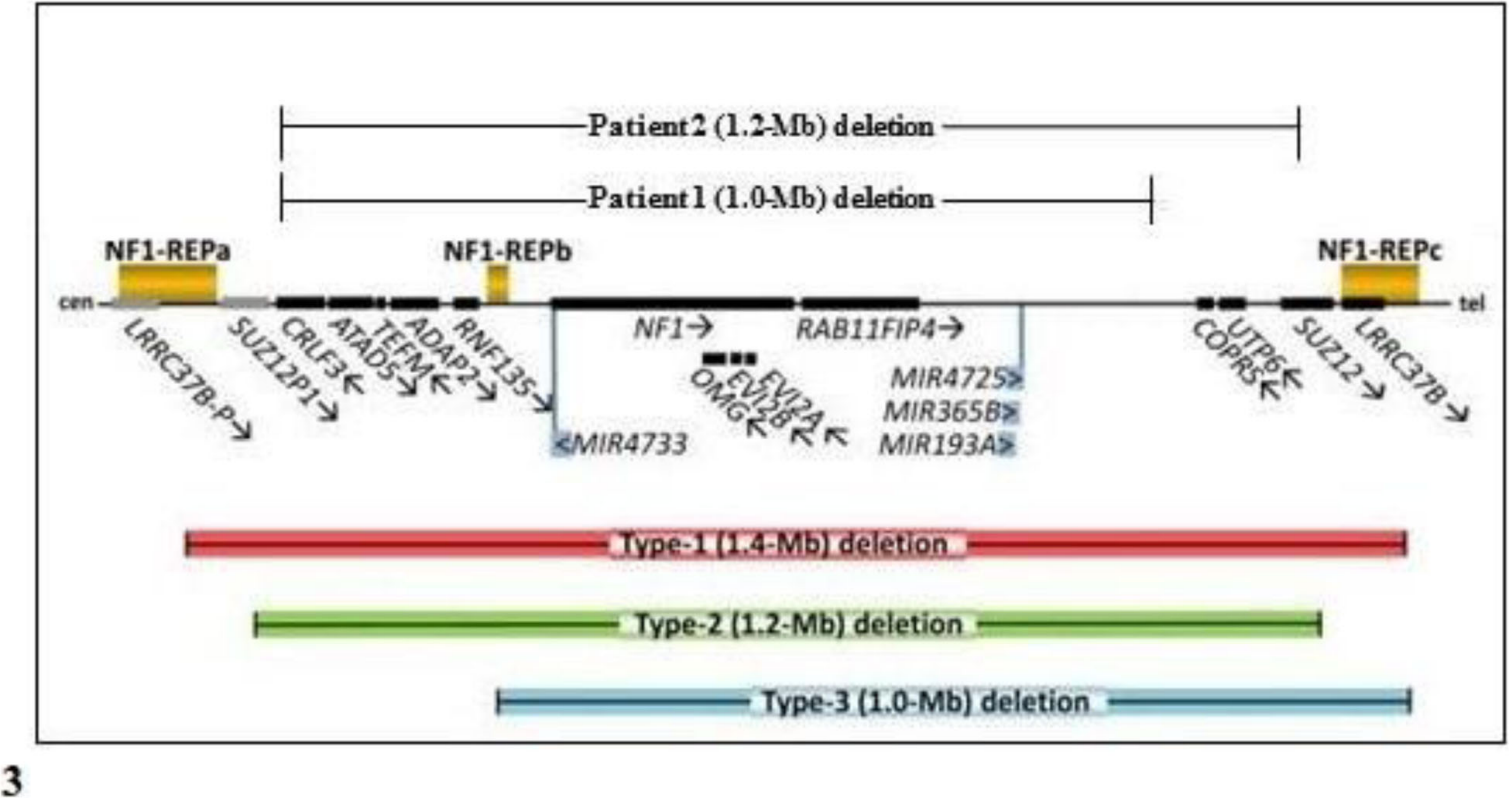
*Schema* of the genome region at 17q11.2 harboring the *NF1* gene and its flanking genes

The deletions of our probands cannot be classified as type 1, 2 or 3, because the breakpoints do not harbor within the low-copy repeats which are recurrent for such deletions (NF1-REPa, b and c), but outside of them (Fig. 3). Particularly, present deletions do not or partially encompass genes typically involved in such rearrangements (i.e. *CRLF3*, *SUZ12*, *LRRC37B* in the case of type-1 and 2), as well as affect genes that are usually spared (i.e. *CRLF3*, *ATAD5*, *TEFM*, *ADAP2* and *RNF135* in type-3) (Fig. 3, modified from Kehrer-Sawatzki et al. [2]).

a-CGH profiles show the three types of recurrent (typical) deletions (type-1, 2 and 3) included within the low-copy repeats (NF1-REPa and c), compared with our Patient 1 and 2 atypical deletions. *Cen* centromeric direction, *tel* telomeric direction.

Both children were clinically evaluated according to the criteria of National Institutes of Health (NIH) for NF1 [7], revised by Gutmann et al. [8]. They fulfilled two criteria (≥6 CALs and axillary/inguinal freckling for both) of the seven established for NF1 diagnosis (including also ≥2 CNFs/SCNFs or 1 plexiform neurofibroma, typical bone lesions, ≥2 Lisch nodules of the iris, optic nerve glioma, an affected first degree relative).

The association between wide *NF1* deletions and a phenotype which is more severe than the NF1 general population, presenting a higher prevalence of learning disabilities, dysmorphic features and increased tumor risk, has been reported [9] and it is consistent with our experience. Specifically, type 1 microdeletion phenotype includes facial dysmorphic features, overgrowth/tall-for-age stature, cognitive delay, scoliosis, bone cysts, large hands and feet, hyperflexibility of joints and muscular hypotonia [2]. The clinical manifestations are related to both the number and type of deleted genes, which in turn has an influence on the genotype-phenotype interaction. Indeed, 17q11.2 microdeletion has been considered as a contiguous gene syndrome, in which the involvement of some of the genes contiguous to *NF1* may modify the phenotype of patients [3]. This may explain the wide clinical variability among microdeletion patients including the two patients here described, which are located at the extremes of the phenotypic spectrum. Comparing the clinical features of our two patients with type-1 *NF1* deletions, a partial phenotypic overlap may be observed. In particular, facial dysmorphisms, neurodevelopmental delay, hypotonia, cardiovascular defects (only in Patient 2), hands/feet anomalies, and kyphoscoliosis (only in Patient 1). Conversely, overgrowth/tall-for-age stature and high tumor load (significantly more frequent in non-mosaic type-1 and 2) are currently absent in our probands, although they cannot be excluded over time. As described above, Patient 1 phenotype is milder than Patient 2 (Table 3).

**Table 3:** Comparison of the clinical and genetic features in both patients

	Patient 1	Patient 2
Sex/ first examination age (years)	F/ 4	F/ 2 and 8 months
*Café-au-lait* spots	15	25
Axillary freckling	+ (Fig. 1)	+
Lisch nodules	1, in the left eye	–
Craniofacial dysmorphic features	high-arched palate	broad forehead dysplasic and low-set ears with thick helix synophris receding orbital roof with exophthalmos hypertelorism depressed nasal bridge bulbous nose malar hypoplasia long and prominent philtrum thick lips
*Pterygium colli*	–	+
Thoracic abnormalities	–	*Pectus excavatum* wide-spaced nipples supernumerary areola (Fig. 2)
Abdominal wall abnormalities	–	*diastasis recti abdominis* with prominent abdomen (Fig. 2)
Oral abnormalities	malocclusion	–
Bone abnormalities	severe kyphoscoliosis bilateral calcaneovalgus foot	short hands/feet clinodactyly of the 5th finger deep palmar creases bilateral *genu valgum* and*pes plano-valgus* (Fig. 2)
Muscular abnormalities	hypotonia/muscular hypotrophy	–
Intellectual disability	–	mild intellectual disability
Cardiovascular defects	–	supravalvular pulmonary stenosis
Developmental delay	speech impairment	global developmental delay
Brain MR abnormalities	–	corpus callosum hypoplasia T2 hyperintensities near the fourth ventricle periventricular hyperintensities hyperintense nodule in the left thalamus
Genetic test result (CGHa – FISH confirmation)	17q11.2 deletion (1 Mb) - partially *CRLF3*, *ATAD5*, *TEFM*, *ADAP2*, *RNF135, OMG, EVI2B, EVI2A, RAB11FIP4* (position 29,124,299 to 30,151,654)	17q11.2 deletion (1.2 Mb) - partially *CRLF3*, *ATAD5*, *TEFM*, *ADAP2*, *RNF135, OMG, EVI2B, EVI2A, RAB11FIP4*, *COPRS*, *UTP6* and partially *SUZ12 (*position 29,124,299 to 30,326,958*)*

Haploinsufficiency of genes within the critical interval could modify the phenotype of present children. To estimate the likely effect of the deleted genes, the probability of loss-of-function (LoF) intolerance should be considered, differentiating genes in LoF intolerant or tolerant [2]. Specifically, 5 genes (*CRLF3*, *ATAD5*, *RAB11FIP4*, *SUZ12* and *LRRC37B*) were predicted to be LoF intolerant [2], which means that they could have clinical consequences if present in one copy [5]; 4 of them (2 partially) are deleted in our subjects.

*RNF135* (ring finger protein 135, MIM:611358), encodes an E3 ubiquitin ligase. This protein contains a ring finger domain, which may be involved in protein-protein and protein-DNA interactions. The gene, expressed in many different tissues (including the cortex and cerebellum), is deleted in both probands. It could be responsible for the dysmorphic facial features of our patients, in agreement with literature data [2]. Its haploinsufficiency may also contribute to the reduced cognitive abilities observed in *NF1* microdeletion patients [2], and then be related to the specific developmental profile of our children. Furthermore, its mutations have been recently documented to be linked to overgrowth and glioblastoma cells in vivo and in vitro, as well as autism, suggesting for patients carrying its deletion, an even more individualized and careful neuropsychiatric and oncologic follow-up.

Similarly, also abnormalities of *OMG* (oligodendrocyte myelin glycoprotein, MIM:164345), deleted in both of our subjects, seem to be involved in autism spectrum disorders, schizophrenia and intellectual disability [10]. Its transcribed protein plays a key role in the early stages of brain development, likely regulating neurogenesis. Indeed, it belongs to myelin-associated inhibitor proteins (MAIPs) which are central nervous system regeneration inhibitors. Since MAIPs are involved in regulating synapses, abnormal expression of these proteins may cause intellectual disability and other brain disorders [2].

Mutations of *ADAP2* (ArfGAP with dual PH domains 2, MIM:608635) may contribute to the cardiovascular malformations noted in patients with *NF1* microdeletions [11, 12]. The gene is highly expressed during early stages of cardiac embryogenesis. The protein encoded by this gene binds beta-tubulin and increases the stability of microtubules. Specifically, cytoskeletal defects of myocytes, due to such altered proteins, may lead to the cardiovascular malformations observed in *NF1* microdeletion patients. Although deleted in both probands, cardiac involvement given by supravalvular pulmonary stenosis is present only in Patient 2. This could be explained since *SUZ12* and *UTP6*, expressed during the development of human heart and deleted only in Patient 2, may contribute either cooperatively or additively to the cardiomyopathy.

As suggested by definition, atypical large *NF1* deletions do not have recurrent breakpoints and are heterogeneous in size and genes deleted [2]. However, the extent of the deletion may be important in the context of genotype-phenotype relationships in such patients. Indeed, our study suggests that these subjects may exhibit most of the clinical features reported in germline type-1 *NF1* deletions. A severe form of the disease is not limited, then, to type-1 deletions, but it may also occur in non-mosaic atypical ones, depending on the extent of the rearrangement and the genes deleted.

Patient 1 shows a neurodevelopmental profile characterized by language disorder without cognitive impairment, and mild craniofacial dysmorphisms (high-arched palate). We propose a correlation between the smaller number of genes/regulatory elements of such deletion and milder clinical manifestations. Therefore, although the clinical characteristics of *NF1* microdeletion patients are usually more severe, this particular form of neurofibromatosis should not be excluded “a priori” even in the presence of patients who show a mild clinical picture (Patient 1), especially in pediatric age, when many of the typical features of the syndrome (i.e. CNFs or SCNFs, MPNST, etc. …) are not yet manifest. Conversely, the more severe clinical picture of Patient 2 may be due to involvement of the genes *COPRS*, *UTP6* and *SUZ12* (the latter predicted to be LoF intolerant). *COPRS* (coordinator of PRMT5 and differentiation stimulator) is widely expressed in human tissues, mostly in testis and brain [13], and has been associated with overgrowth as well as learning disabilities and dysmorphic features of *NF1* microdeletion patients. It encodes an adaptor protein binding to protein-arginine methyltransferase 5 (PRMT5) and to hystone H4. Such COPRS-PRMT5 complex regulates cell differentiation, and is then involved in tumorigenesis. *UTP6* (*UTP6* small subunit processome component) and *SUZ12* (*SUZ12* polycomb repressive complex 2 subunit, MIM:606245) may be associated with congenital heart defects [11], and together with *COPRS* they may contribute to the increased tumour risk associated with *NF1* microdeletion [14]. However, although their haploinsufficiency could exert additive negative effects on the phenotype of Patient 2, no conclusions can be drawn yet with regards to their role in the specific phenotype. The limited number of microdeletion patients together with the imprecise definition of their boundaries, especially in atypical deletions, make it quite difficult to establish reliable genotype-phenotype correlations [15]. Then, genetic analysis, not always performed in the past, is actually a diagnostic step in characterizing a patient with or suspected of NF1. In case of microdeletion, a-CGH is an important tool to finely define deletions. Although NF1 diagnosis is still based on clinical criteria, information obtained from genetic analysis can be an important instrument to plan a more individualized clinical follow-up.

Phenotypical variability is observed in *NF1* microdeletion patients, even in cases of identical germline deletions [16]. Hence, the phenotype associated with *NF1* microdeletions is likely to be influenced by genetic elements such as the expression variations of non-deleted genes and/or the presence of feature-specific modifier genes. In addition, epigenetic factors such as the expression of the wild-type alleles of the genes present in one copy could play a role. Environmental factors may also interact with both of them. An extended comparative analysis of a larger number of age-matched patients with additional overlapping features may allow a more precise clinical and genomic characterization [17]. This is also useful to achieve a better genotype-phenotype correlation.

## Supplementary information

**Additional file 1.** Timelines of the clinical cases.

## Data Availability

The datasets used and/or analyzed during the current study are available from GC on reasonable request.

## Abbreviations

a-CGH: array comparative genomic hybridization
CALs: *Café-au-lait* spots
CNFs/SCNFs: Cutaneous/subcutaneous neurofibromas
EEG: Electroencephalogram
FISH: Fluorescence in situ hybridization
MLPA: Multiplex ligation-dependent probe amplification
MPNST: Malignant peripheral nerve sheath tumors
MRI: Magnetic resonance imaging
NF1: Neurofibromatosis type 1
NIH: National Institutes of Health
OFC: Occipitofrontal circumference
PCR: Polymerase chain reaction
PPVT-III: Peabody Picture Vocabulary Test-III
US: Ultrasound
WPPSI: Wechsler Preschool and Primary Scale of Intelligence
