# Supplementary material for: NF1 microdeletion syndrome: case report of two new patients
Source: Ital J Pediatr. 2019 Nov 8;45:138. doi: 10.1186/s13052-019-0718-7 (PMC6839219; doi:10.1186/s13052-019-0718-7)
Supplement: Supplementary file 1 — Additional file 1. Timelines of the clinical cases. [file 13052_2019_718_MOESM1_ESM.doc]

**Patient 1**

Clinical genetic evaluation

First neuropsychiatric evaluation: deficits in language-related abilities

Brain MRI and US heart evaluation: normal findings

4 years old

6 years old

Hypotonia/muscular hypotrophy, dysgraphia and hyperactivity

Diagnosis of *NF1* deletion

FISH confirmation

No mutations

Testing of the parents

FISH on buccal swab DNA

*de novo* origin

No somatic mosaicism

**Patient 2**

First neurodevelopmental examination: global developmental delay

18 months old

EEG: normal

US heart evaluation: mild valvar pulmonary stenosis

Sequencing of the genes *PTPN11*, *RAF1*, *BRAF1*, *MEK1/2*, *KRAS*, *SOS1*, *SHOC2*: no alterations

Normal plasma amino acid pattern, plasma and urine levels of glycosaminoglycans, acylcarnitine profile and urine organic acids

Clinical genetic evaluation

Brain MRI: corpus callosum hypoplasia, T2 hyperintensities near the fourth ventricle, periventricular hyperintensities and a hyperintense nodule in the left thalamus

Genetic testing

Molecular analysis of *NF1* and *SPRED1*: no alterations

MLPA: diagnosis of *NF1* deletion

High resolution a-CGH: 1.2 Mb deletion

PCR: breakpoint positions

FISH confirmation

Neuropsychiatric evaluation (WPPSI): mild intellectual disability

2 years old

2 years and 8 months old

6 years old
